# Supplementary material for: DsbA-L deficiency in T cells promotes diet-induced thermogenesis through suppressing IFN-γ production
Source: Nat Commun. 2021 Jan 12;12:326. doi: 10.1038/s41467-020-20665-4 (PMC7804451; doi:10.1038/s41467-020-20665-4)
Supplement: Supplementary file 1 — Supplementary Information [file 41467_2020_20665_MOESM1_ESM.pdf]

Supplementary information

DsbA-L Deficiency in T cells Promotes Diet-induced Thermogenesis through Suppressing IFN-γ Production

Haiyan Zhou, Xinyi Peng, Jie Hu, Liwen Wang, Hairong Luo, Junyan Zhang, Yacheng Zhang, Guobao Li, Yujiao Ji, Jingjing Zhang, Juli Bai, Meilian Liu, Zhiguang Zhou, Feng Liu

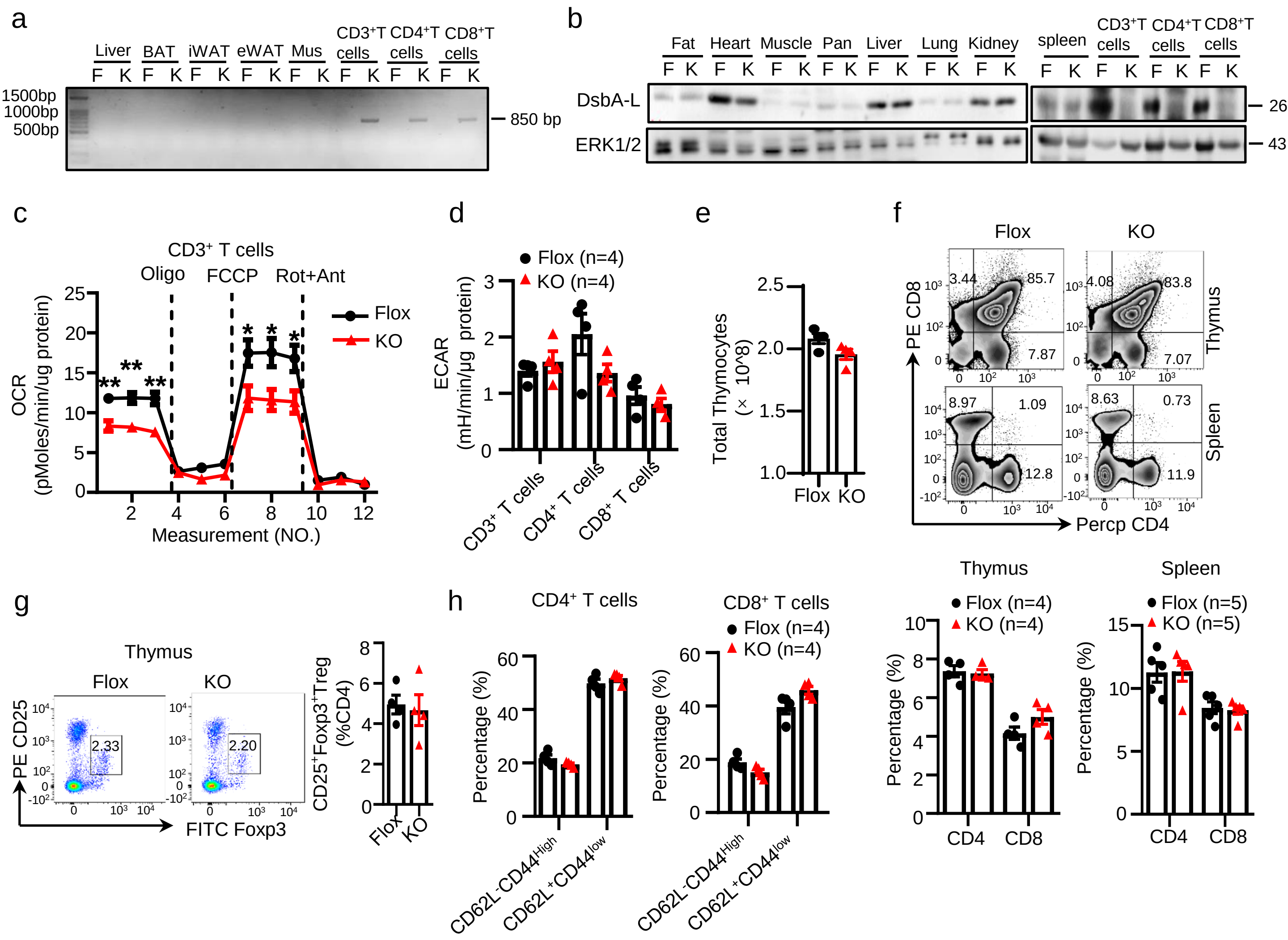

Supplementary Figure 1 T cell-specific DsbA-L deficiency has no effect on T cell development. (Related to Fig.2).

(a) DNA was extracted from tissues and cells of DsbA-L$^{CD4\text{-}KO}$ mice and control littermates. PCR was performed to confirm successful loxP site recombination mediated by Cre with primers as follows: Fw: CTGGATGGCTTCTGTTAGAG; Rv: GGATGGAGGACCGTGTCATC. Mus: muscle.

(b) Protein levels of DsbA-L in tissues and cells collected from 8-week-old DsbA-L$^{CD4\text{-}KO}$ mice and control littermates. Pan: pancreas.

(c) OCR of activated CD3$^+$ T cells was measured under basal conditions and in response to indicated drugs. (n=4/group). Oligo, oligomycin; Rot, rotenone; Ant, antimycin.

(d) ECAR of activated CD3$^+$, CD4$^+$, and CD8$^+$ T cells were measured under basal conditions. (n=4/group)

(e) Total thymocytes in 8-week-old DsbA-L$^{CD4\text{-}KO}$ mice and control littermates. (n=4/group)

(f) Frequencies of CD4$^+$ and CD8$^+$ T cells in thymus (n=4/group) and spleen (n=5/group) of 8-week-old DsbA-L$^{CD4\text{-}KO}$ mice and control littermates.

(g) Treg development in the thymus of DsbA-L$^{CD4\text{-}KO}$ mice and control littermates. (n=4/group)

(h) Frequencies of CD62L$^+$CD44^{Low} naive T cells and CD62L$^-$CD44^{High} memory T cells in CD4$^+$ and CD8$^+$ T cells in spleen of 8-week-old DsbA-L$^{CD4\text{-}KO}$ mice and control littermates. (n=4/group)

Data shown are representative of three independent experiments. All data are presented as mean ± SEM. Statistical values $p < 0.05$ (*), $p < 0.01$ (**), $p < 0.001$ (***) are determined by two-tailed unpaired Student's t test. Source Data are provided as a Source Data File.

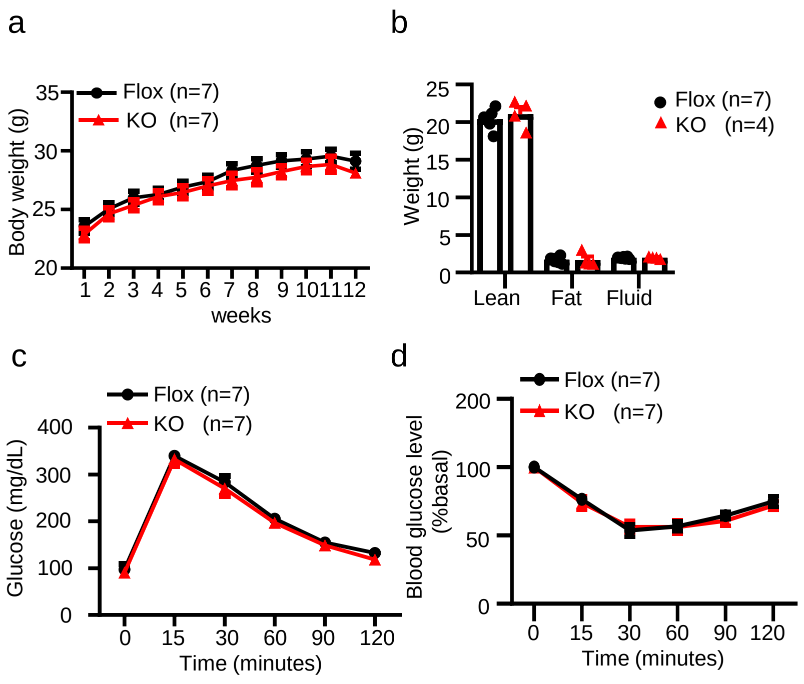

Supplementary Figure 2 Metabolic phenotypes of DsbA-L$^{CD4\text{-}KO}$ mice and control littermates under ND or HFD feeding conditions (Related to Fig.3).

(a) Body weight gain of DsbA-L$^{CD4\text{-}KO}$ mice (n=7) and control littermates (n=7) during ND feeding.

(b) Body composition of DsbA-L$^{CD4\text{-}KO}$ mice (n=7) and control littermates (n=4) fed a ND for 12 weeks.

(c) Glucose tolerance test was performed on DsbA-L$^{CD4\text{-}KO}$ mice (n=7) and control littermates (n=7) fed a ND for 12 weeks.

(d) Insulin tolerance test was performed on DsbA-L$^{CD4\text{-}KO}$ mice (n=7) and control littermates (n=7) fed a ND for 12 weeks.

Data shown are representative of three independent experiments. All data are presented as mean ± SEM. Source data are provided as a Source Data File.

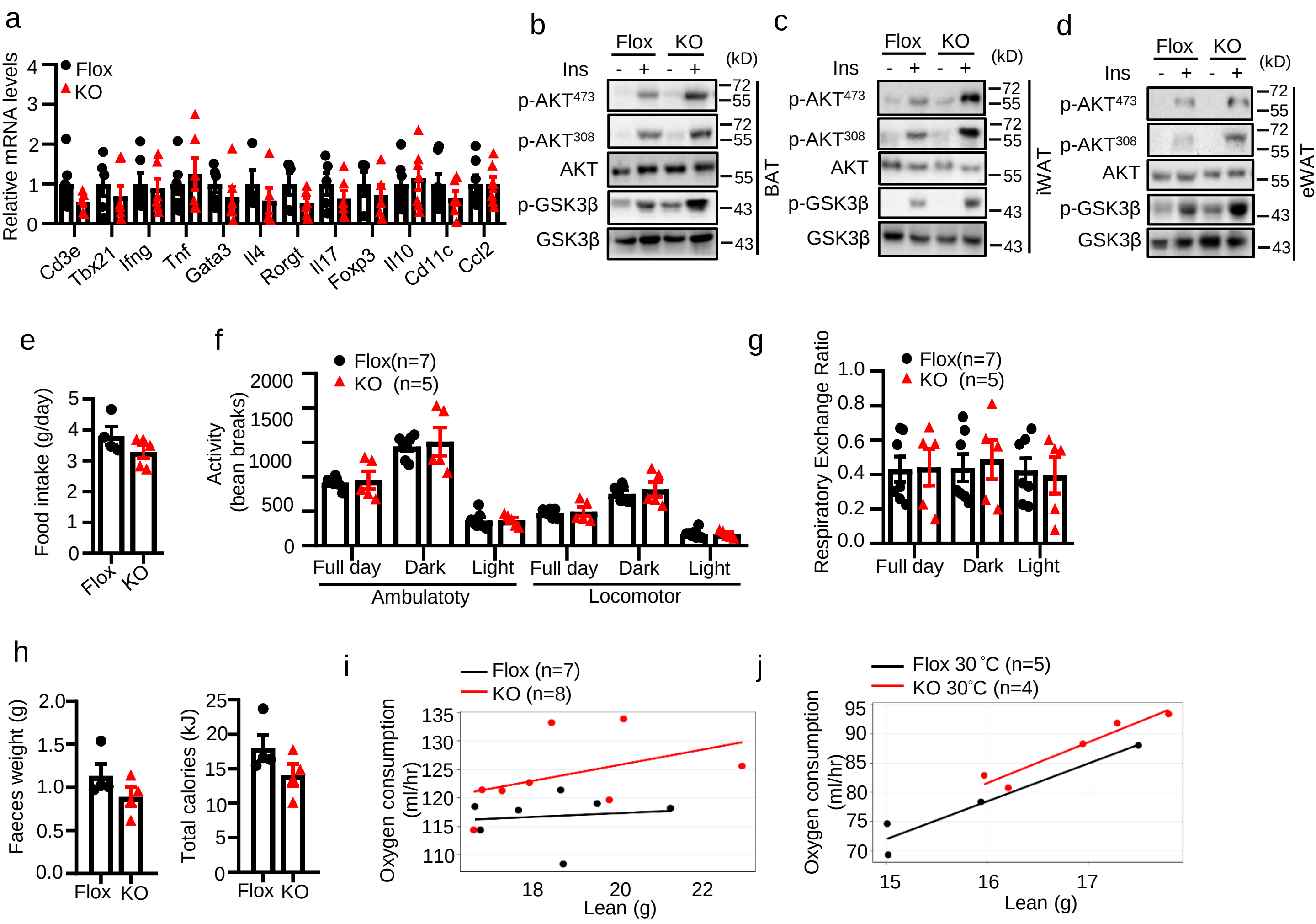

Supplementary Figure 3 Metabolic Phenotypes of DsbA-L^{CD4-KO} mice and control littermates under HFD feeding conditions (Related to Fig.3 and Fig.4).

(a) Inflammation in the eWAT of DsbA-L^{CD4-KO} mice (n=5-7) and control littermates (n=4-7) fed a HFD for 12 weeks.

(b-d) Insulin signaling was examined in BAT (b), iWAT (c), and eWAT (d) of DsbA-L^{CD4-KO} mice and control littermates fed a HFD for 12 weeks. Ins, insulin.

(e) Food intake in DsbA-L^{CD4-KO} mice (n=5) and control littermates (n=4) fed a HFD for 12 weeks.

(f) Physical Activity in DsbA-L^{CD4-KO} mice (n=5) and control littermates (n=7) fed a HFD for 12 weeks.

(g) RER in DsbA-L^{CD4-KO} mice (n=5) and control littermates (n=7) fed a HFD for 12 weeks.

(h) Dried faeces weights and total faeces energy in DsbA-L^{CD4-KO} mice (n=4) and control littermates (n=4) fed a HFD for 12 weeks.

(i) ANCOVA analyses of average VO_2 (ml/hr) by lean mass in DsbA-L^{CD4-KO} mice and control littermates fed a HFD for 5 weeks.

(j) ANCOVA analyses of average VO_2 (ml/hr) by lean mass in DsbA-L^{CD4-KO} mice and control littermates which were fed a HFD for 5 weeks and then tested under thermoneutral conditions (30°C).

Data shown are representative of three independent experiments. All data are presented as mean ± SEM. Source data are provided as a Source Data File.

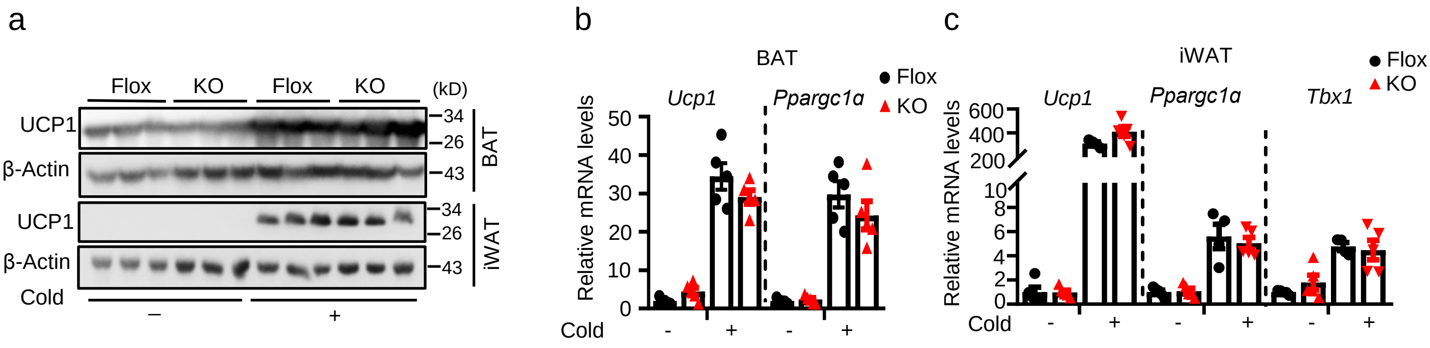

Supplementary Figure 4 DsbA-L deficiency in T cells has no significant effect on CIT (Related to Fig.4).

(a) Western blot analyses of UCP1 levels in the BAT and iWAT of DsbA-L$^{CD4\text{-}KO}$ mice and control littermates after cold stimulation.

(b and c) mRNA levels of thermogenic and beige marker genes in the BAT (n=5/group) (b) and iWAT (n=4-5/group) (c) of DsbA-L$^{CD4\text{-}KO}$ mice and control littermates after cold stimulation.

Data shown are representative of three independent experiments. All data are presented as mean ± SEM. Source data are provided as a Source Data File.

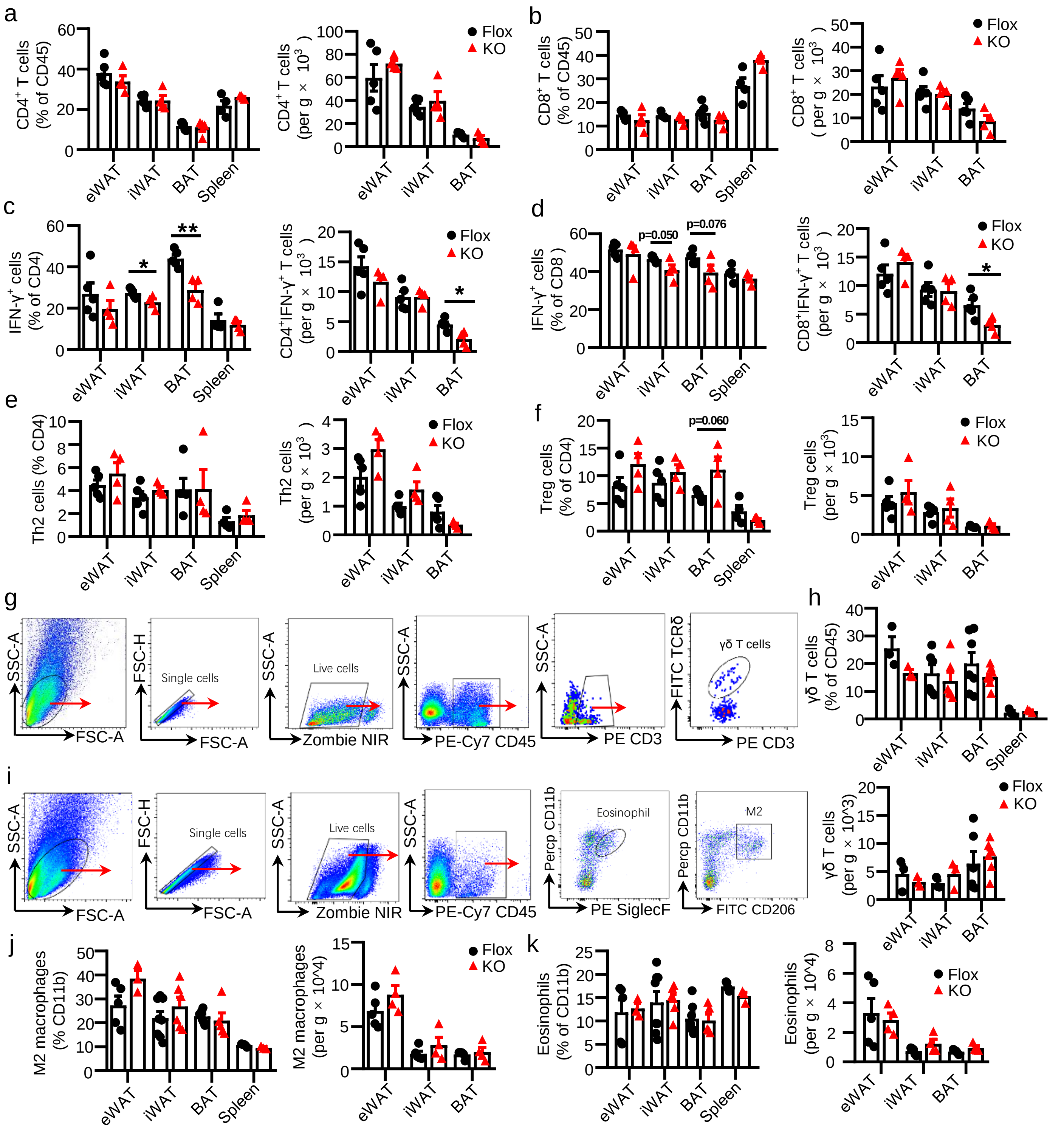

Supplementary Figure 5 T cell phenotypes in the adipose tissues of DsbA-L$^{CD4\text{-}KO}$ mice and control littermates fed a HFD for 5 weeks and 12 weeks (Related to Fig.5).

(a and b) Quantification of the frequencies and total numbers of CD4$^+$ (a) and CD8$^+$ (b) T cells in the eWAT, iWAT, BAT, and spleen of DsbA-L$^{CD4\text{-}KO}$ mice (n=4) and control littermates (n=5) fed a HFD for 12 weeks.

(c-f) Quantification of the frequencies and total numbers of CD4$^+$IFN-γ$^+$ Th1 cells (c), CD8$^+$IFN-γ$^+$ T cells (d), Th2 cells (e), and Treg cells (f) in the eWAT, iWAT, BAT, and spleen of DsbA-L$^{CD4\text{-}KO}$ mice (n=4) and control littermates (n=5) fed a HFD for 12 weeks.

(g) Gating strategies of γδ T cells in the adipose tissues.

(h) Quantification of the frequencies and total numbers of γδ T cells in the eWAT , iWAT, BAT, and spleen of DsbA-L$^{CD4\text{-}KO}$ mice and control littermates fed a HFD for 5 weeks. (n=3-9/group)

(i) Gating strategies of M2 macrophages and eosinophils in the adipose tissues.

(j and k) Quantification of the frequencies and total numbers of M2 macrophages (j) and eosinophils (k) in the eWAT, iWAT, BAT, and spleen of DsbA-L$^{CD4\text{-}KO}$ mice and control littermates fed a HFD for 5 weeks. (n=3-8/group)

Data shown are representative of three independent experiments. All data are presented as mean ± SEM. Statistical values $p < 0.05$ (*), $p < 0.01$ (**), $p < 0.001$ (***) are determined by two-tailed unpaired Student's t test. Source data are provided as a Source Data File.

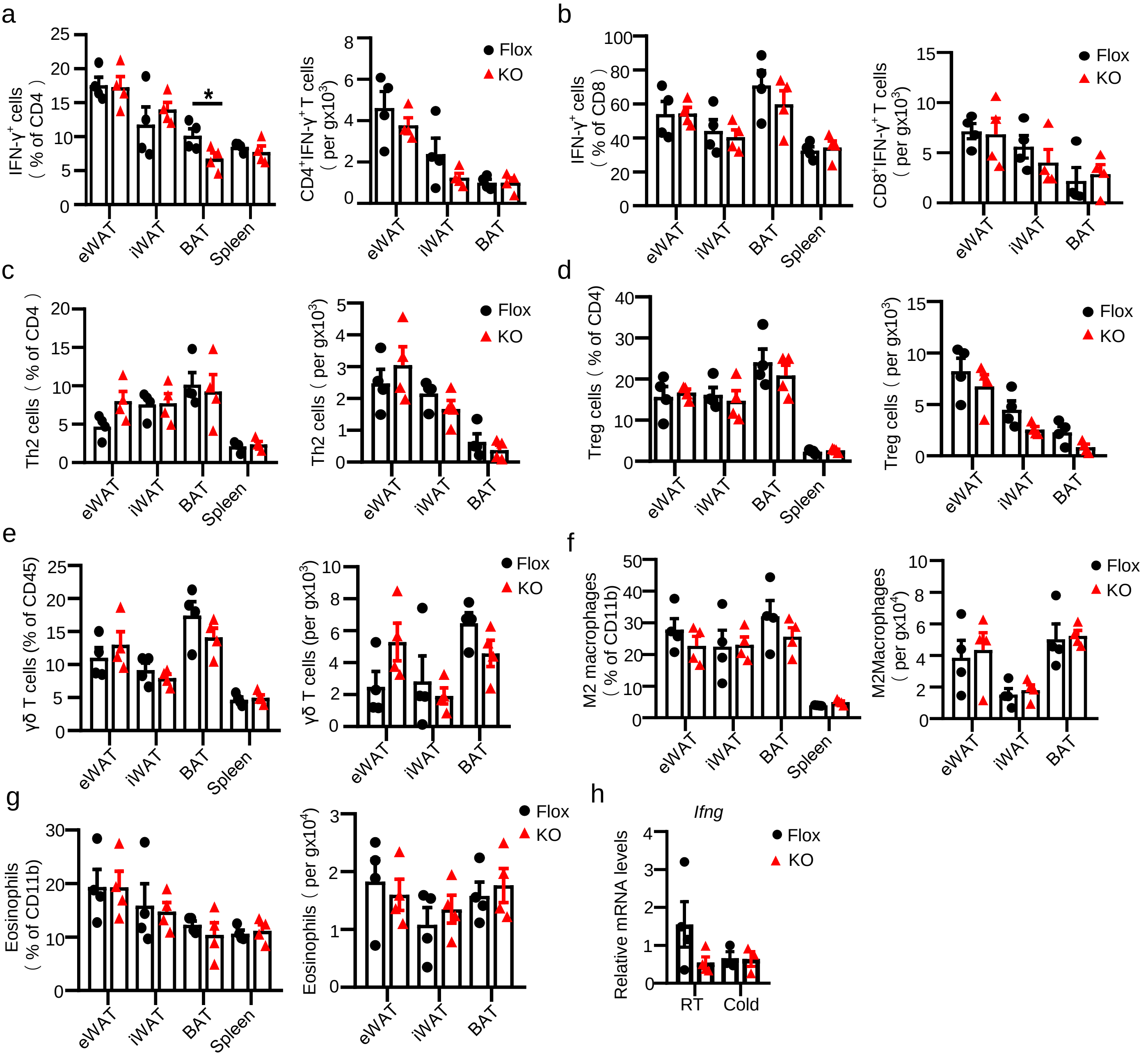

Supplementary Figure 6 T cell phenotypes in the adipose tissues of DsbA-L$^{CD4\text{-}KO}$ mice and control littermates after cold stimulation (Related to Fig.5)

8-week-old DsbA-L$^{CD4\text{-}KO}$ mice (n=4) and control littermates (n=4) were exposed to cold stimulation for 1 week.

(a-e) Quantification of the frequencies and total numbers of CD4$^+$IFN-γ$^+$ Th1 cells (a), CD8$^+$IFN-γ$^+$ T cells (b), Th2 cells (c), Treg cells (d), and γδ T cells (e) in the eWAT, iWAT, BAT, and spleen.

(f and g) Quantification of the frequencies and total numbers of M2 macrophages (f) and eosinophils (g) in the eWAT, iWAT, BAT, and spleen.

(h) IFN-γ mRNA levels in the BAT of 8-week-old DsbA-L$^{CD4\text{-}KO}$ mice and control littermates with or without cold stimulation. RT: room temperature.

Data shown are representative of three independent experiments. All data are presented as mean ± SEM. Statistical values $p < 0.05$ (*), $p < 0.01$ (**), $p < 0.001$ (***) are determined by two-tailed unpaired Student's t test. Source data are provided as a Source Data File.

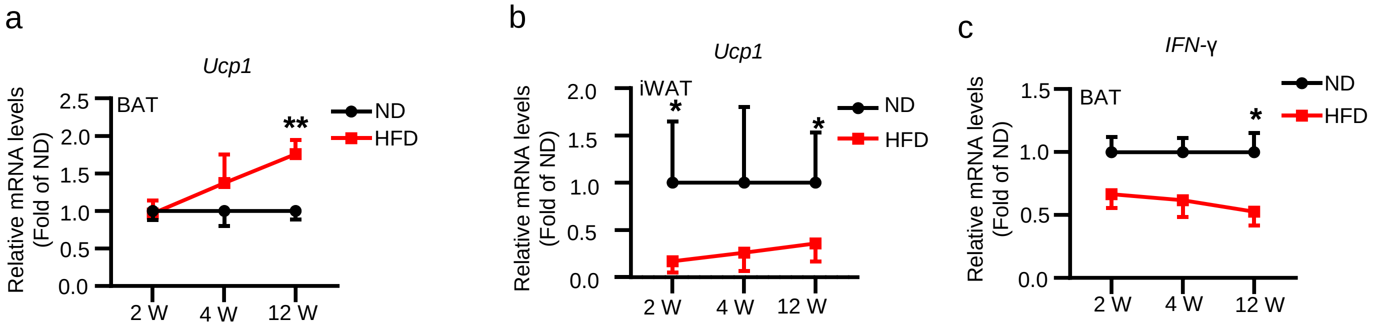

Supplementary Figure 7 IFN-γ expression negatively correlates with UCP1 expression in BAT after HFD feeding (Related to Fig.6).

Six-week-old C57BL/6 mice (n=6/group) were fed a ND or HFD for 2, 4, or 12 weeks.

(a) *Ucp1* mRNA levels in BAT of mice fed a ND or HFD.

(b) *Ucp1* mRNA levels in iWAT of mice fed a ND or HFD.

(c) IFN-γ mRNA levels in BAT of mice fed a ND or HFD.

Data shown are representative of at least three independent experiments. All data are presented as mean ± SEM. Statistical values $p < 0.05$ (*), $p < 0.01$ (**), $p < 0.001$ (***) are determined by two-tailed unpaired Student's t test. Source data are provided as a Source Data File.

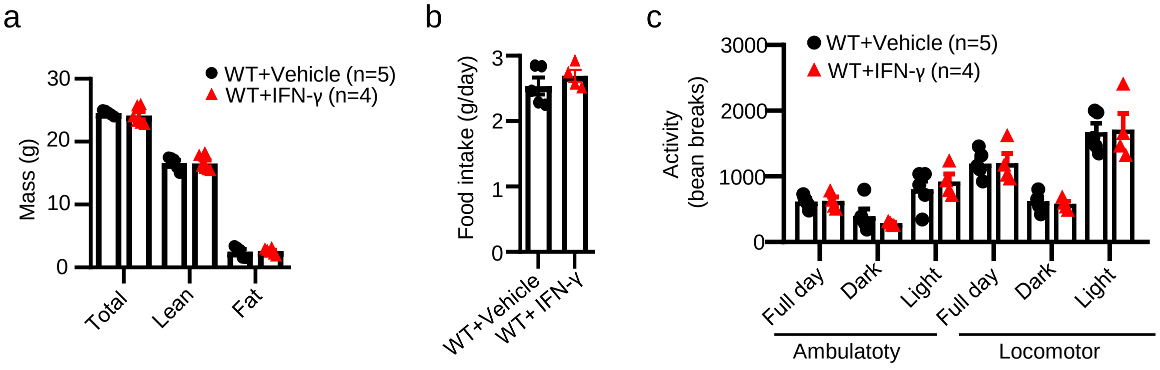

Supplementary Figure 8 Metabolic phenotypes of wild-type mice fed a HFD for 5 weeks and treated with or without IFN-γ for the last week (Related to Fig.7).

Body weights and fat mass (a), food intake (b), as well as physical activity (c) of wild-type mice fed a HFD for 5 weeks with (n=4) or without IFN-γ (n=5) administration for the last week.

Data shown are representative of three independent experiments. All data are presented as mean ± SEM.

Source data are provided as a Source Data File.

Supplementary Table 1: Primer pairs sequences for qPCR analysis

Gene	Primers	Sequences
DsbA-L	Forward primer	GCAGTTGCGGCCCACTT
	Reverse primer	TGGTTGGTTTCCGCTGTCTT
Prdm16	Forward primer	TGAGGAAGCATTTGAAGTTAAAG
	Reverse primer	GTTCTTAGCCTGCCTGTAC
Ppargc1a	Forward primer	CCGAAGACACTACAGGTTCCATAG
	Reverse primer	GGGAGGGAGAGAGGAGAGAGG
Ucp1	Forward primer	AAGACAGAAGAGCATAGCATTCAC
	Reverse primer	CCAGTCATACACTCCCACCTC
Cebpb	Forward primer	CCAAGGCCAAGGCCAAGAAGAC
	Reverse primer	ACAAGTTCCGCAGGGTGCTGAG
Cidea	Forward primer	TGCTCTTCTGTATCGCCCAGT
	Reverse primer	GCCGTGTTAAGGAATCTGCTG
Dio2	Forward primer	CAGTGTGGTGCACGTCTCCAATC
	Reverse primer	TGAACCAAAGTTGACCACCAG
Atg1	Forward primer	GCTGTGGAATGAGGACATAGGA
	Reverse primer	GCATAGTGAGTGGCTGGTGAA
Hsl	Forward primer	TGTGTCAGTGCCTATTCAG
	Reverse primer	GAACAGCGAAGTGTCTCT
Acc1	Forward primer	TGCCACCACCTTATCACTATGTA
	Reverse primer	CCTGCCTGTCTCCATCCA
Fasn	Forward primer	TCGTCTATACCACTGCTTACTAC
	Reverse primer	ACACCACCTGAACCTGAG
Il2	Forward primer	GCAGGCCACAGAATTGAAAGA
	Reverse primer	TGCCGCAGAGGTCCAAGT
Il5	Forward primer	TGCACTTGAGTGTTCTGACTCTCA
	Reverse primer	TGTGCTCATGGGAATCTCCAT
Il6	Forward primer	CCACGGCCTTCCCTACTTC
	Reverse primer	TTGGGAGTGGTATCCTCTGTGA
Il10	Forward primer	GATGCCCCAGGCAGAGAA
	Reverse primer	CACCCAGGGAATTCAAATGC
Il13	Forward primer	TTGAGGAGCTGAGCAACATCAC
	Reverse primer	CCATGCTGCCGTTGCA
Ccl2	Forward primer	GTCTGTGCTGACCCCAAGAAG
	Reverse primer	TGGTTCCGATCCAGGTTTTTA
Ifng	Forward primer	TTGGCTTTGCAGCTCTTCCT
	Reverse primer	TGACTGTGCCGTGGCAGTA
Tgfb1	Forward primer	GCAGTGGCTGAACCAAGGA
	Reverse primer	AGCAGTGAGCGCTGAATCG
Cd3e	Forward primer	CTCAGAAGCATGATAAGCAC
	Reverse primer	CAGAGTGATACAGATGTCAAC

Gata3	Forward primer	GACCCGAAACCGGAAGATGT
	Reverse primer	GCGCGTCATGCACCTTTT
Foxp3	Forward primer	GGCCCTTCTCCAGGACAGA
	Reverse primer	GGCATGGGCATCCACAGT
Rorgt	Forward primer	TGCGACTGGAGGACCTTCTAC
	Reverse primer	TCACCTCCTCCCGTGAAAAG
Il17	Forward primer	CCTGGCGGCTACAGTGAAG
	Reverse primer	TTTGGACACGCTGAGCTTTG
Cd11c	Forward primer	TGGGCCTGTCCCTTGCT
	Reverse primer	ACAGTAGGACCACAAGCCAACA
Tbx21	Forward primer	ACCTGTTGTGGTCCAAGTTCAA
	Reverse primer	GCCGTCCTTGCTTAGTGATGA
16s rRNA	Forward primer	CCGCAAGGGAAAGATGAAAGAC
	Reverse primer	TCGTTTGGTTTCGGGGTTTC
mt-Atp6	Forward primer	CACTATGAGCTGGAGCCGTAATT
	Reverse primer	GAAGTGGGCAAGTGAGCTTTTT
Erra	Forward primer	CGGTGTGGCATCCTGTGA
	Reverse primer	CTCCCCTGGATGGTCCTCTT
Aco2	Forward primer	GCCCAGATGGCTATGCTACAG
	Reverse primer	CGCAGGTCTTTCTCACCCC
Atp5a1	Forward primer	TCTCCATGCCTCTAACACTCG
	Reverse primer	CCAGGTCAACAGACGTGTCAG
Sdhb	Forward primer	CTGAATAAGTGCGGACCTATGG
	Reverse primer	AGTATTGCCTCCGTTGATGTTC
Cpt1a	Forward primer	AGATCAATCGGACCCTAGACAC
	Reverse primer	CAGCGAGTAGCGCATAGTCA
Cpt2	Forward primer	CAGCACAGCATCGTACCCA
	Reverse primer	TCCCAATGCCGTTCTCAAAAT
Mcad	Forward primer	ATGCCTGTGATTCTTGCTGGA
	Reverse primer	ACATCTTCTGGCCGTTGATAAC
Acox1	Forward primer	CCGCCACCTTCAATCCAGAG
	Reverse primer	CAAGTTCTCGATTTCTCGACGG
Ppara	Forward primer	TACTGCCGTTTTCACAAGTGC
	Reverse primer	AGGTCGTGTTCACAGGTAAGA
Tbx1	Forward primer	GGCAGGCAGACGAATGTTC
	Reverse primer	TTGTCATCTACGGGCACAAAG
Tmem26	Forward primer	ACCCTGTCATCCCACAGAG
	Reverse primer	TGTTTGGTGGAGTCCTAAGGTC
Klhl13	Forward primer	GGAACTGTGTATGGAGGAGTGATG
	Reverse primer	GTTCCTCGGAAATGGTTGCC
Adrb3	Forward primer	GACTACAGACCATAACCAACGTG
	Reverse primer	CCTGGTGGCATTACGAGGA

Pde4a	Forward primer	TCAGCGAGGAGGACACTCTTC
	Reverse primer	TTTCTGCCTCCAAGCTGACA
Pde4b	Forward primer	CAAACAAGGTAAGGACACCTCTT
	Reverse primer	TGGTAGCAAGGTACGAGCAAA
Pde4d	Forward primer	TTTCTTCCCGCAGCATTCA
	Reverse primer	GTCTGCTTGTTCCAACTGTCTGA
Th	Forward primer	TCTCCTTGAGGGGTACAAAACC
	Reverse primer	ACCTCGAAGCGCACAAAGT
β-actin	Forward primer	GTTGGTTGGAGCAAACATC
	Reverse primer	CTTATTTCATGGATACTTGGAATG
