# Supplementary material for: DsbA-L deficiency in T cells promotes diet-induced thermogenesis through suppressing IFN-γ production
Source: Nat Commun. 2021 Jan 12;12:326. doi: 10.1038/s41467-020-20665-4 (PMC7804451; doi:10.1038/s41467-020-20665-4)
Supplement: Supplementary file 3 — Reporting Summary [file 41467_2020_20665_MOESM3_ESM.pdf]

nature research

Corresponding author(s): Feng Liu, Haiyan Zhou

Last updated by author(s): Nov 23, 2020

Reporting Summary

Nature Research wishes to improve the reproducibility of the work that we publish. This form provides structure for consistency and transparency in reporting. For further information on Nature Research policies, see our Editorial Policies and the Editorial Policy Checklist.

Statistics

For all statistical analyses, confirm that the following items are present in the figure legend, table legend, main text, or Methods section.

n/a	Confirmed	
☐	☒	The exact sample size (*n*) for each experimental group/condition, given as a discrete number and unit of measurement
☐	☒	A statement on whether measurements were taken from distinct samples or whether the same sample was measured repeatedly
☐	☒	The statistical test(s) used AND whether they are one- or two-sided<br>*Only common tests should be described solely by name; describe more complex techniques in the Methods section.*
☐	☒	A description of all covariates tested
☐	☒	A description of any assumptions or corrections, such as tests of normality and adjustment for multiple comparisons
☐	☒	A full description of the statistical parameters including central tendency (e.g. means) or other basic estimates (e.g. regression coefficient) AND variation (e.g. standard deviation) or associated estimates of uncertainty (e.g. confidence intervals)
☐	☒	For null hypothesis testing, the test statistic (e.g. *F*, *t*, *r*) with confidence intervals, effect sizes, degrees of freedom and *P* value noted<br>*Give P values as exact values whenever suitable.*
☒	☐	For Bayesian analysis, information on the choice of priors and Markov chain Monte Carlo settings
☒	☐	For hierarchical and complex designs, identification of the appropriate level for tests and full reporting of outcomes
☒	☐	Estimates of effect sizes (e.g. Cohen's *d*, Pearson's *r*), indicating how they were calculated

Our web collection on statistics for biologists contains articles on many of the points above.

Software and code

Policy information about availability of computer code

Data collection	BD FACS Diva software v9.0 and BD Canto II (flow cytometry), Applied Biosystem 7900HT Real-Time PCR System (qPCR), Oxymax-CLAMS and Oxymax Systems (metabolic cages), Minispec Body Composition Analyzer LF50 (body composition), Zeiss lsm780 confocal microscope system (immunofluorescence), Seahorse XF24 extracellular flux analyzer (Oxygen consumption rates).
Data analysis	FlowJo V10 (flow cytometry),GraphPad Prism 8 .0 (data analysis), ImageJ 1.44 (imaging analysis), CalR (R language Version 4.0.3)

For manuscripts utilizing custom algorithms or software that are central to the research but not yet described in published literature, software must be made available to editors and reviewers. We strongly encourage code deposition in a community repository (e.g. GitHub). See the Nature Research guidelines for submitting code & software for further information.

Data

Policy information about availability of data

All manuscripts must include a data availability statement. This statement should provide the following information, where applicable:

- Accession codes, unique identifiers, or web links for publicly available datasets
- A list of figures that have associated raw data
- A description of any restrictions on data availability

We have included a data availability statement in our manuscript. The data that support the findings of this study are available from the corresponding author upon reasonable request. Raw data of figures are provided as a Source Data file.

Field-specific reporting

Please select the one below that is the best fit for your research. If you are not sure, read the appropriate sections before making your selection.

- [x] Life sciences
- [ ] Behavioural & social sciences
- [ ] Ecological, evolutionary & environmental sciences

For a reference copy of the document with all sections, see nature.com/documents/nr-reporting-summary-flat.pdf

Life sciences study design

All studies must disclose on these points even when the disclosure is negative.

Sample size	Sample size was not predetermined but we performed experiments with group sizes based on existing published literature of similar experiments. For animal experiments, $n \geq 4$ was chosen based on the previous publications in the field (Meilian Liu et al., 2014, Cell metabolism). For experiments other than those involving animals, $n \geq 3$ was chosen based on the previous publications in the field (Tuo Deng et al., 2013, Cell metabolism) and also because this size is necessary to calculate statistical significances.
Data exclusions	In qPCR data, samples were rarely excluded when the RNA quality was poor or the expression of the house keeping genes (internal controls) in a particular sample differed from the average of the other samples by more than 2 Ct value. The exclusion criteria (the expression of the house keeping genes) was pre-established.
Replication	All experimental findings were reliably reproduced for three times and all replication attempts were successful.
Randomization	Animals were randomly assigned to experimental and control groups. In particular, the authors measured mouse body weight before the beginning of the experiment to make sure there is no weight difference between control and experimental groups.
Blinding	Investigators were blinded to group allocation during data collection. Investigators were blinded for analysis of histological specimens and metabolic tests. In experiments without subjective estimation like flow cytometry and qPCR, investigators were unblinded since no bias would be introduced by the investigators.

Reporting for specific materials, systems and methods

We require information from authors about some types of materials, experimental systems and methods used in many studies. Here, indicate whether each material, system or method listed is relevant to your study. If you are not sure if a list item applies to your research, read the appropriate section before selecting a response.

Materials & experimental systems

n/a	Involved in the study	
☐	☒	Antibodies
☐	☒	Eukaryotic cell lines
☒	☐	Palaeontology and archaeology
☐	☒	Animals and other organisms
☒	☐	Human research participants
☒	☐	Clinical data
☒	☐	Dual use research of concern

Methods

n/a	Involved in the study	
☒	☐	ChIP-seq
☐	☒	Flow cytometry
☒	☐	MRI-based neuroimaging

Antibodies

Antibodies used	Western Blotting:<br>Anti-UCP1 (ab10983,abcam), Anti-PGC1-a (ab54481,abcam), Anti-p-PKA substrate (#9621,cell signaling), Anti-p-Creb (#9198,cell signaling), Anti-total-Creb (#9197, cell signaling),Anti-p-DRP1 (#3455, cell signaling), Anti-total DRP1 (#5391, cell signaling), Anti-p-GSK3β (9323T,cell signaling), Anti-total GSK3β (9315S,cell signaling), Anti-beta-Actin (MABT825 ,Sigma), Anti-ERK1/2 (#4695S,Cell Signaling), Anti-DsbA-L (homemade), Anti-OPA1 (27733-1-AP, Proteintech), Anti-MFN2 (12186-1-AP, Proteintech).<br>Flow cytometry:<br>The following antibodies were perchased from BD biosciences:FVS-520 (564407), APC-Cy7-CD45 (557659), Percp-CD4 (553052), APC-CD62L (561919), PE-Cy7-CD44 (560569), Alexa fluor 647-GATA3 (560068). The following antibodies were perchased from Biolegend: Zombie NIR (423105), PE-Cy7-CD45 (103114), PE-CD3 (100206), PE-CD4 (100511), PE-CD25 (102007), APC-CD8 (100711), PE-Cy7-CD8 (100721), PE-CD8 (100708), APC-IFN-γ (505810), Alexa fluor 647-Foxp3 (320013), Percp-Cy5.5-CD11b (101228), APC-F4/80 (123115), FITC-CD206 (141710). The following antibodies were perchased from ebioscience: PE-Siglec-F (12-1702-82), FITC-γδTCR (11-5711-81), FITC-Foxp3 (11-5773-82).<br>Immunohistochemistry: Anti-UCP1 (ab10983,abcam)<br>Others: Hamster Anti-Mouse CD3e (BD biosciences, Clone 145-2C11, 553057), secondary anti-hamster antibody (Biolegend, Clone Poly4055, 405501)
Validation	Our homemade anti-DsbA-L antibody was validated by western blot analyses of from mouse adipose tissues collected from fat specific-DsbA-L knockout mice and control mice (Bai et al., PNAS,2017). We also validated the antibody in DsbA-L-knockout T cells. All commercial antibodies were carefully selected for their desired applications. Validation details on the manufacturer's websites indicate that these antibodies could detect proteins in mouse species and are appropriate for their relevant application.<br>Anti-UCP1 (ab10983,abcam) is recommended by the manufacturer for detection of mouse UCP1 by western blotting and immunohistochemistry.<br>Anti-PGC1-a (ab54481,abcam) is recommended by the manufacturer for detection of PGC1 alpha in mouse brown adipose tissue whole cell extract .<br>Anti-p-PKA substrate (#9621,cell signaling) is recommended by the manufacturer for detection of mouse endogenous protein phosphorylated by PKA.<br>Anti-p-CREB (Ser133) (87G3) detects endogenous levels of CREB only when phosphorylated at serine 133.<br>Anti-total-Creb (#9197, cell signaling) detects endogenous levels of total CREB-1 protein. The antibody does not cross-react with other ATF/CREB family members.<br>Anti-p-GSK-3β (Ser9) (9323T,cell signaling) is recommended by the manufacturer for detection of mouse endogenous GSK-3β only when phosphorylated at serine 9.<br>Anti-GSK-3β (Ser9) (9315S,cell signaling) is recommended by the manufacturer for detection of mouse endogenous total GSK-3β protein.<br>Anti-p-DRP1 (#3455, cell signaling) detects endogenous levels of DRP1 only when phosphorylated at Ser616.<br>Anti-DRP1 (#5391, cell signaling) recognizes endogenous levels of total DRP1 protein.<br>Anti-beta-Actin (MABT825 ,Sigma) is validated for use in Western Blotting, Immunocytochemistry for the detection of beta-Actin.<br>Anti-ERK1/2 (#4695S,Cell Signaling) detects endogenous levels of total p44/42 MAP kinase (Erk1/Erk2) protein. The antibody does not cross-react with JNK/SAPK or p38 MAP kinase.<br>Anti-OPA1 (27733-1-AP, Proteintech) targets OPA1 in WB, IP, IHC, IF, ELISA applications and shows reactivity with Human, rat, mouse samples.<br>Anti-MFN2 (12186-1-AP, Proteintech) targets MFN2 in WB, IP, IHC, IF, ELISA applications and shows reactivity with human, mouse, rat samples.<br>FVS-520 (564407) is useful for discrimination of viable from non-viable mammalian cells in multicolor flow cytometric applications.<br>Zombie NIR (423105) is an amine reactive fluorescent dye that is non-permeant to live cells, but permeant to the cells with compromised membranes. Thus, it can be used to assess live vs. dead status of mammalian cells.

Eukaryotic cell lines

Policy information about cell lines

Cell line source(s)	The brown adipocyte cell line BFC was a generous gift from Dr. Jiandie Lin (Univ. of Michigan) .
Authentication	The cell line was authenticated as described in the paper (Uldry M et al., Cell metabolism,2006) and has been used in our previous paper (Meilian Liu et al., Cell metabolism, 2014). The cell line was authenticated by lipid accumulation on adipocyte differentiation, cell morphology by microscopy, and thermogenic gene expression by qPCR analyses.
Mycoplasma contamination	The BFC cell line was tested negative for mycoplasma contamination.
Commonly misidentified lines (See ICLAC register)	No commonly misidentified cell line was used.

Animals and other organisms

Policy information about studies involving animals; ARRIVE guidelines recommended for reporting animal research

Laboratory animals	Mus musculus<br>Wild-type mice, C57BL/6J background, 8 week-old male mice were used.

	DsbA-L flox/flox mice, C57BL/6J background, 8 week-old male mice were used.<br>CD4-Cre mice, C57BL/6J background, 8 week-old male mice were used to cross with DsbA-L flox/flox mice.<br>DsbA-L-CD4-KO mice, C57BL/6J background,8 week-old male mice were used.
Wild animals	This study does not involve wild animals.
Field-collected samples	This study does not involve field-collected samples.
Ethics oversight	All animal studies were performed under a protocol approved by the Central South University Animal Care and Use Committee.

Note that full information on the approval of the study protocol must also be provided in the manuscript.

Flow Cytometry

Plots

Confirm that:

- [x] The axis labels state the marker and fluorochrome used (e.g. CD4-FITC).
- [x] The axis scales are clearly visible. Include numbers along axes only for bottom left plot of group (a 'group' is an analysis of identical markers).
- [x] All plots are contour plots with outliers or pseudocolor plots.
- [x] A numerical value for number of cells or percentage (with statistics) is provided.

Methodology

Sample preparation	Adipose tissues were carefully excised, minced, and digested with 1.5 g/L type 2 collagenase (Sigma-Aldrich) for 25 min at 37° C, with shaking. Digested cells were filtered with a 100 µm nylon screen, washed, and centrifuged for 5 min to pellet the stromal vascular fractions (SVFs) from floating mature adipocytes. To detect the expression of surface molecules, SVFs were first incubated with an anti-Fc receptor (Biolegend, San Diego, CA) to reduce nonspecific binding of antibodies, followed by incubation with the indicated antibodies for 20-30 min at 4°C. For analysis of transcription factor expression, surface labeled cells were fixed, permeabilized and stained with indicated antibodies for 40-50 min at 4 °C with Transcription Factor Buffer Set (BD Biosciences, San Jose, CA) according to the manufacturer's instructions. For analysis of intracellular IFN-γ, cells were stimulated with PMA (50 ng/ml; Beyotime, Shanghai, China) and ionomycin (750 ng/ml; Millipore, Darmstadt, Germany) for 6 h with the addition of Brefeldin A (10 µg/ml; Beyotime, Shanghai, China). Cells were harvested, washed, fixed, permeabilized with the Fixation/Permeabilization Solution Kit (BD Biosciences, San Jose, USA) and stained with the APC-IFN-γ antibody. Appropriate fluorescein-conjugated, isotype-matched, irrelevant mAbs were used as negative controls.
Instrument	BD Canto II (BD Biosciences, San Jose, USA)
Software	Data were collected using BD FACS Diva software and were analyzed by FlowJo V10 (BD Biosciences, San Jose, USA).
Cell population abundance	No post-sorting analysis was done.
Gating strategy	Preliminary FSC/SSC gates of the starting cell population of SVFs were set according to the FSC/SSC gates of lymphocytes in the spleen samples. Dead cells and doublets were removed by dead-cell dye staining and FSC-A/FSC-H gating, respectively. Positive and negative populations were selected by single-positive control samples and isotype-matched negative control samples.

- [x] Tick this box to confirm that a figure exemplifying the gating strategy is provided in the Supplementary Information.
